# Accumulation Potential of Lead and Cadmium Metals in Maize (*Zea mays* L.) and Effects on Physiological-Morphological Characteristics

**DOI:** 10.3390/life15020310

**Published:** 2025-02-17

**Authors:** Ümit Elik, Zeynep Gül

**Affiliations:** 1 Artuklu District Directorate of Agriculture and Forestry, 47060 Mardin, Türkiye; umitelik@tarimorman.gov.tr; 2 Plant Production Application and Research Center, Atatürk University, 25100 Erzurum, Türkiye

**Keywords:** maize, cadmium, lead, heavy metal, plant growth, accumulation

## Abstract

Phytoremediation stands at the forefront of modern environmental science, offering an innovative and cost-effective solution for the remediation of heavy-metal-contaminated soils through the natural capabilities of plants. This study aims to investigate the effects of lead (Pb) and cadmium (Cd) metals on plant growth (e.g., seedling height, stem diameter, fresh and dry weight), physiological properties (e.g., tissue relative water content, tissue electrical conductivity), and biochemical parameters (e.g., chlorophyll content, superoxide dismutase (SOD), catalase (CAT), peroxidase (POD) enzyme activities) of maize compared to the control group under greenhouse conditions at the Atatürk University Plant Production Application and Research Center. The results show that plant height decreased by 20% in the lead (Pb3000) application and by 42% in the cadmium (Cd300) application compared to the control group. The highest Pb dose (Pb3000) caused a 15% weight loss compared to the control, while the highest Cd dose (Cd300) caused a weight loss of 63%. The accumulation rates of heavy metals in soil, roots, and aboveground parts of plants indicated that maize absorbed and accumulated more Cd compared to Pb.

## 1. Introduction

Heavy metals are significant pollutants that disrupt the environmental balance, inhibiting the growth and development of living organisms [1]. Beyond protecting the environment and natural resources from pollution, remediating existing contaminated areas plays a crucial role in preventing further environmental degradation. Contemporary issues such as the irresponsible use of pesticides and chemicals, urbanization, and industrial activities contribute to environmental pollution and heavy metal accumulation [2]. Heavy metals mixed with soil, air, and water reach living beings in various ways and threaten human, animal, and plant life [3]. Since heavy metals are not biodegradable, it is difficult to remove the pollution they cause in the soil [4]. Heavy metals occur naturally at certain levels in plants and other living things [3]. As they have various structural functions in the metabolism of living things, some structural disorders may occur in living organisms and many physiological problems may develop in their deficiency [5]. In addition, when heavy metals are ingested in excessive doses, they can cause the development of various reactions that can threaten the living conditions of living organisms. This condition is generally referred to as heavy metal poisoning or toxicity [6].

It is known that heavy metal pollution in soil affects about 235 million hectares of arable land worldwide [7]. As with all types of pollution, most plants are affected by heavy metal pollution [8]. The heavy metal uptake potential of each plant is different. The internal mechanisms of heavy metal absorption are described in the literature as “accumulation, symptom, and exclusion” [9]. In addition, many studies have shown significant differences in plant metal uptake, even between genotypes of species [10]. Plants generally take up heavy metals present in the soil as ions through their roots. The negative effect of heavy metal stress on the plant depends not only on the type of metal and its concentration in the soil. Depending on the genetics of different species, it is also significantly related to their physiological behavior [11]. This shows that each plant species has different tolerance thresholds for heavy metal stress. Researchers have found that some plant species can tolerate toxic compounds in high-metal soils [12,13].

The physical and chemical methods used to remove heavy metals, considered to be the most critical soil pollutants, are costly or time consuming. Instead, natural and economical alternatives are emphasized [14]. The phytoremediation method, also defined as healing or greening, is the use of hyperaccumulator plants to remove pollution. Hyperaccumulator plants can accumulate 50 to 500 times more metal in their bodies (leaves, branches, and stems) than the metal content of the soil [7]. The fact that hyperaccumulator plants have the structure to accumulate these metals in their roots, leaves, and stems allows the soil to be cleaned of metals by harvesting the plant. It is known that the tolerance of plants to heavy metal stress varies depending on the type of plant, the heavy metal, and the duration of the stress [15]. It is therefore important to know what damage is caused by the type and concentration of heavy metal damage and in which tissues it accumulates in plants.

Lead (Pb) is one of the most toxic inorganic metal pollutants worldwide and a persistent environmental pollutant [16]. Pb is produced by many industrial processes and then mixes with soil, water, and atmosphere [17,18]. Pb released into the environment is easily taken up by plants and is included in the soil-plant-human system and the food chain and accumulates in the human body [19]. The toxicity of Pb, one of the non-essential ions in plants, causes inhibition of seed germination, restriction of plant growth, and reduction of crop yield [20]. After Pb penetrates the roots, most of it is retained in the root tissues, while a small part is transported to the aboveground parts of the plant [21]. Enzymatic reactions have a toxic effect on many processes in plant morphology, such as chlorophyll biosynthesis and membrane permeability [20,22]. Studies have been conducted to understand the mechanism of Pb tolerance and phytoremediation in many plants. However, Pb uptake, transport, accumulation, and tolerance differ among species [23].

One of the most important of the environmental pollutants known as heavy metals is cadmium [24]. As it has higher solubility and mobility in water than other metals, it can easily mix with air, plants, and water resources [25]. It reduces the yield of plants and negatively affects the quality and quantity of products [26]. Cd is phytotoxic even at low concentrations; it can be easily transported to plants from contaminated soils and is toxic to plants, animals, and humans [27]. Plants grown in areas with high Cd concentrations may threaten human and animal health if they accumulate Cd in their edible parts [28]. Approximately 3% of the Cd in Cd-containing soils can be transmitted to humans through the consumption of rice grown in such soils [29]. It causes growth retardation in plants, suppression of photosynthesis, change (decrease) in plant–water relations, uptake and distribution of macro- and micronutrients, disruption of stomatal movements, and negativities in enzymatic activities and protein metabolism [30,31]. Cd accumulated in the plant damages the morphology, physiology, and biochemical aspects of the plant through passive or active means [32].

Increasing Pb and Cd pollution has recently become a growing concern in the world. The accumulation of these metals, particularly in agricultural lands, causes significant yield losses in crops such as wheat, barley, and maize, which has a major impact on human nutrition [33,34]. In the statement published in 2022 by the US Agency for Toxic Substances and Disease Registry (ATSDR), Pb and Cd are ranked 2nd and 7th, respectively, among 275 priority hazardous pollutants [35].

Native to the Americas, maize is now one of the most widely cultivated crops in the world [36]. It is cultivated on 206 million hectares worldwide, mainly in the temperate zone [28]. Turkey ranks 23rd in world maize production [37]. While 61% of global maize production is used as animal feed, 13% is used for human consumption as the main source of energy and carbohydrates [38].

Therefore, any potential contaminant associated with maize poses a significant risk to human health. Most of the previous studies have investigated the tolerance of maize plants under stress conditions caused by Pb and Cd metals [39,40,41,42]. Previous research has shown that genetic factors influence the tolerance level, even within the same plant [43,44,45]. This study aims to scientifically establish the quality and safety of silage production in the face of increasing heavy metal pollution, by examining the physiological and morphological effects of Pb and Cd metal toxicity and the accumulation potential in the early-maturing *Dekalp 6442* variety, known for being well-suited to the region’s climatic conditions. The research contributes new and original insights to the literature by revealing the tolerance and accumulation capacity of maize, a valuable forage crop evaluated as silage in the region, against heavy metal stress. Additionally, the lack of previous studies on the heavy metal accumulation potential of a silage variety in the region makes this research unique and significant.

## 2. Materials and Methods

### 2.1. Plant Material

This research started with the greenhouse phase in January and February 2022. After the plants reached harvest maturity, the analysis process continued with greenhouse and laboratory measurements and ended in April. The study was carried out as a pot experiment in the greenhouse of Atatürk University Plant Production Application and Research Center. Maize (*Zea mays* L. cv. *Dekalp 6442*) seeds were used in the research. *Dekalp 6442* variety is an early silage maize seed suitable for Erzurum and similar ecologies (short vegetation period), reaching harvest maturity in 90 days.

### 2.2. Heavy Metal Treatments and Experimental Design

In the study, 2 L pots were filled with a mixture of garden soil, peat, and sand (2:1:1 *v*:*v*:*v*), and the prepared medium (the soil is loamy soil containing 31% sand, 36% silt, and 33% clay) was 15 kg ha^−1^ N, 7.5 kg ha^−1^ P_2_O_5_, and 18 kg ha^−1^ K_2_O [46]. The randomized plots experimental design comprised 7 applications, 3 repetitions, and 5 pots from each repetition, for a total of 105 (7 × 3 × 5 = 105) pots. In order to pollute the soil, different doses of cadmium (100, 200, and 300 mg kg^−1^ CdSO_4_·8H_2_O) and lead (1000, 2000, and 3000 mg kg^−1^ PbNO_3_) were added [47,48]. After mixing and watering, the medium was incubated for 3 weeks. After the incubation period, 7 maize seeds were planted in each pot. After emergence, the plants were thinned to four plants per pot.

**Figure 1:**
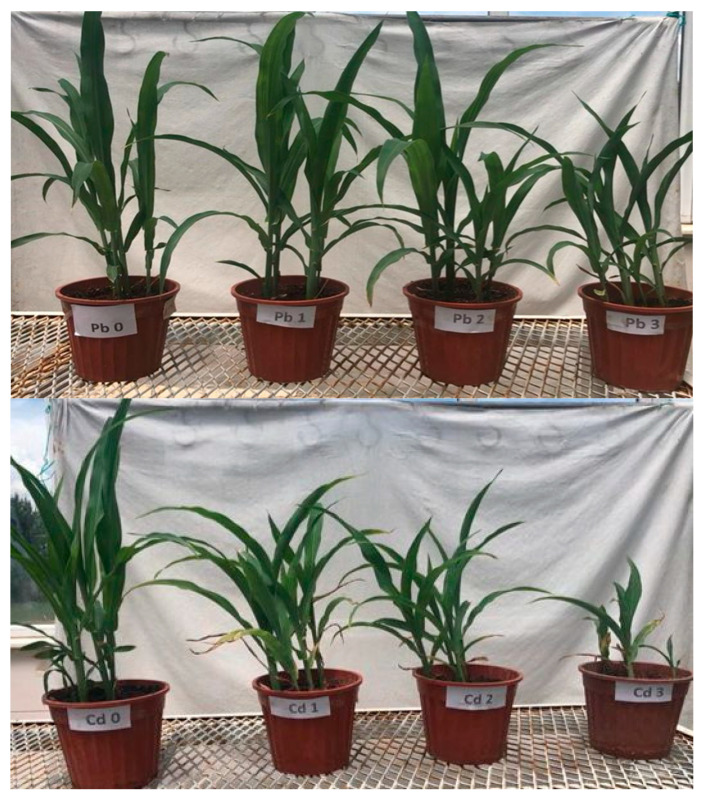
Effects of Pb and Cd applications on the growth of maize seedlings (V6 period).

The study was completed in 50 days when the plants reached the harvest stage (V6) [49] (Figure 1). At the end of the experiment, the following measurements were made while the plants were in or out of the pot. Plant growth parameters such as plant height, plant/root fresh and dry weight (70 °C for 48 h), stem diameter, and leaf number were determined.

### 2.3. Observations and Measurements

#### 2.3.1. Plant Growth Parameters

In this study, plant height, stem diameter, number of leaves, plant fresh and dry weight, and root fresh and dry weight were determined at the end of study. The chlorophyll reading value of leaves was determined by the SPAaD-502 device (SPAD-502, Konica Minolta Sensing, Inc., Tokyo, Japan). Leaf area in a plant was detected with a CI-202 Portable Area Meter (CID, Inc., Camas, WA, USA) [50].

#### 2.3.2. Leaf Relative Water Content (LRWC) and Electrolyte Conductivity (EC)

For LRWC analysis, leaf samples from two plants for each treatment were weighed (FW). Then, water was added, and after waiting for 24 h, the turgid weights of the leaf samples were determined (TW). After this, the samples were dried at 70 °C for 48 h and their dry weights (DW) were determined. LRWC was calculated according to Turan et al. [51].

Electrolyte conductivity (EC) was determined on leaf samples taken from the middle leaves of two seedlings. Firstly, the samples were incubated for 24 h in distilled water, and EC1 was measured with an electrical conductivity meter. After the samples were autoclaved at 120 °C for 20 min, EC2 was determined when the solution temperature reached 25 °C. EC value was defined according to EC1/EC2 × 100 formula [51,52].

#### 2.3.3. Catalase (CAT), Peroxidase (POD), and Superoxide Dismutase (SOD) Enzyme Activities

Fresh leaf samples were homogenized in the extraction solution according to the method specified by Liu et al. [53] and Turan et al. [51], and the obtained supernatant was used to determine enzyme activities. CAT activity was determined by the decrease in absorbance of H_2_O_2_ at 240 nm [52]. POD activity of samples was determined at 436 nm and SOD activity at 560 nm with the method of Liu et al. [53] by spectrophotometry.

#### 2.3.4. Hydrogen Peroxide (H_2_O_2_) and Malondialdehyde (MDA)

H_2_O_2_ and MDA content of leaf tissues were determined according to the method of Liu et al. [53]. Approximately 0.3 g of fresh leaf samples was homogenized in a mortar with 5% trichloroacetic acid (TCA) and centrifuged at 4100 rpm at +4 °C for 20 min, and the supernatant was used for H_2_O_2_ and MDA analyses. The MDA content was determined using spectrophotometry at 532 and 600 nm absorbance and defined as mmol kg fw^−1^ with the formula [(Abs532 nm − Abs600 nm)/1.55 × 105] using the extinction coefficient of 155 mM^−1^ cm^−1^. H_2_O_2_ was determined at 390 nm in a spectrophotometer, a standard graph created and defined as mmol kg fw^−1^ [54].

#### 2.3.5. Accumulation Parameters

To determine the heavy metal contents of Cd and Pb in the harvested, dried, and ground maize plant samples, wet combustion was performed by adding a mixture of nitric acid and perchloric acid. The heavy metal content ratios of roots and aerial parts were then determined in the ICP-OES instrument [55]. Soil samples collected and sieved from the plots were prepared according to the principles of the DTPA method proposed by [56]. The Cd and Pb contents of the soils were determined by ICP-OES.

#### 2.3.6. Bioconcentration Factor (BCF) and Translocation Factor (TF)

The bioconcentration factor (BCF) and the translocation factor (TF), defined as the transferability of Cd from soil to crop, were calculated as below [57,58]:

BCF = average pollutant concentration of the root/average pollutant concentration of soil;

TF = average pollutant concentration in the aboveground parts of the plant/average pollutant concentration of the root [54].

#### 2.3.7. Statistical Analysis

In the experiment, a randomized design was used, and the data obtained were analyzed using SPSS 20 software. The data were subjected to analysis of variance (ANOVA), and differences in means were determined by Duncan’s multiple comparison test. The Komogrov–Smirnov test was used for normality analysis, and the Levene test was used for variance homogeneity analysis. The significance level was taken as 0.05.

## 3. Results

### 3.1. Effects of Heavy Metal Applications on the Growth Characteristic

The findings of the study clearly demonstrate the adverse effects of heavy metal application on the growth characteristics of maize plants. Firstly, it was observed that different concentrations of Cd and Pb metals caused significant growth retardation in maize plants. This phenomenon results from the pressure exerted by heavy metal toxicity on plant physiology. Apart from mineral nutrients, roots also take up heavy metal ions present in the soil and transport them to different plant organs. Subsequently, there may be a significant decrease in shoot and root growth under metal stress [59]. The study showed that Cd is more toxic than Pb, exerting more pronounced negative effects on plant growth parameters. Under Cd300 treatment, plant height, fresh weight, dry weight, root fresh weight, and root dry weight values were found to decrease by 42%, 63%, 62%, 56%, and 53%, respectively, compared to the control group (Table 1). This finding suggests that cadmium significantly restricts plant growth through mechanisms such as inhibiting protein synthesis, adversely affecting photosynthesis, and impeding carbohydrate transportation within plant cells [60]. Similar growth retardation was observed with Pb applications. However, Pb was found to be less toxic than Cd, with relatively milder effects on plant growth parameters. In an identical study, Cd, chromium (Cr), and nickel (Ni) had the most negative effects on root and shoot lengths and dry matter accumulation in maize organs. When maize plants were grown in the presence of 36 mg Cd/kg, root length was significantly reduced by 65.3% compared to the untreated control [59]. In the Pb3000 treatments, plant height decreased by 20%, fresh weight by 15%, dry weight by 25%, root fresh weight by 17%, and root dry weight by 10% compared to the control group (Table 1). The study demonstrated that heavy metal applications lead to a reduction in stem diameter and leaf number in maize plants. Specifically, stem diameter decreased by 25% in Pb3000 treatments and by 52% in Cd300 treatments (Table 1). As a matter of fact, Pb metal delays seed germination and reduces seedling growth, germination percentage, germination index, root/shoot length, tolerance level, and dry mass of roots and shoots [61].

### 3.2. Effects of Heavy Metal Applications on Physiological Traits

#### Leaf Relative Water Content (LRWC), Electrolyte Conductivity

The effects of various Pb and Cd levels on the EC and LRWC of maize plants are shown in Table 2. Electrical conductivity (EC) (%) values increased with the effect of the metals under study. While the EC value of the control group was 10.76%, it increased up to 15.22% in lead applications (Pb3000) and 20.93% in cadmium applications (CD300). LRWC values decreased compared to the control group with the effect of metal applications. The LRWC value of the control application was calculated as 81.48%. The highest value in lead application (Pb1000) was determined as 70.58%, and the lowest value (Pb3000) was determined as 62.32%. In cadmium applications, the highest value (Cd100) was determined as 75.40%, and the lowest value (Cd300) was determined as 66.41%. Previous research [49] has stated that low doses of cadmium cause more effective stress on the plant than high doses of lead. The most important reason for the decrease in water and ion uptake of plants under cadmium stress is that it inhibits root growth and development [62]. Our findings are consistent with [63], who stated that Cd increases EC in beans. Similarly, previous researchers have shown that heavy metal stress causes a decrease in the LRWC of various products [64,65]

### 3.3. Effects of Heavy Metal Applications on Biochemical Traits

The effects of Pb and Cd heavy metals on antioxidant enzyme activities in maize plants are summarized in Table 2. Although CAT activity increased in Pb applications, no significant difference was observed between Pb1000 and Pb3000 treatments. However, CAT activity decreased in the Pb2000 treatment. All doses of Cd applications increased CAT activity. For peroxidase (POD) enzyme activity, the control application value was calculated as 8.21 EU/g FW (Table 2). Except for Pb2000, Pb applications had lower values than the control application. In Cd applications, all doses except for Cd300 were higher than the control level. In our study, although SOD activity increased in Pb applications compared to the control group, all applications were in the same statistical group (Table 2). The findings of this study indicate that Pb and Cd contamination has an increasing effect on H_2_O_2_ and MDA levels (Table 2). In the control group, the H_2_O_2_ and MDA values were found to be 98.69 and 1.75 nmol/g, respectively. In the Pb3000 application, the values were 152.57 and 2.46 nmol/g, respectively, while in the Cd300 application, they were 125.68 and 6.46 nmol/g. Similar release of CAT, SOD, etc. in different organs (e.g., shoots and roots) of maize plants grown under stress has been reported by other researchers [66] Increased levels of MDA and antioxidant enzymes indicate the severity of heavy metal stress in maize plants. However, the expression of MDA and antioxidant enzymes is vital because such biomolecules play an important role in stabilizing and protecting the cell membrane from metal damage and consequently enable plants to overcome metal toxicity [59].

### 3.4. Pb and Cd Accumulation

**Figure 2:**
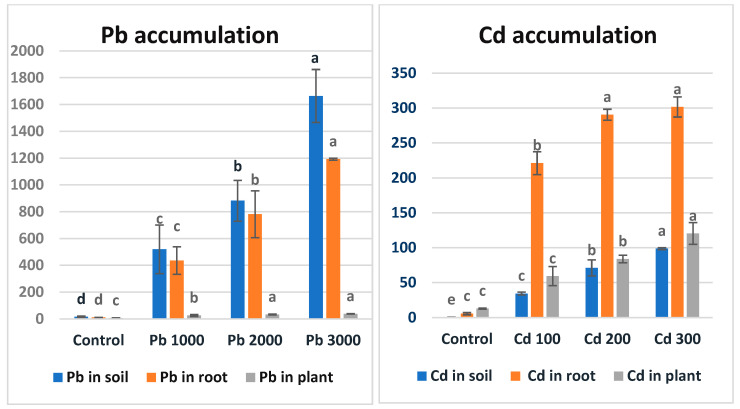
Accumulation values (ppm) of Pb and Cd metals in soil, roots, and plants in maize after the 7-week growth period. There is no statistical difference between the averages shown with the same letter.

The accumulation values of different Pb and Cd applications in soil, roots, and above-ground parts are shown in Figure 2. As seen in Figure 2, the maize plant accumulated lead metal in the order of soil > roots > above-ground parts. The extensive roots of plants absorb lead together with its ions from Pb-contaminated soils [67]. After passive adsorption and accumulation by the roots, they reach the apoplastic pathways and are then transported by the vascular flow system [68]. The adsorbed Pb is mostly stored in the roots, but some of its concentration is transported to the aerial parts of the plants [69]. Endodermal cells act as a physical barrier against Pb transfer, since they reduce Pb translocation through the symplast and apoplast pathways [20]. The researchers found that Pb metal transfer coefficients were significantly lower than those for Cd and Zn [70]. A study examining the accumulation of lead metal in roots, stems, and leaves in tomato, pepper, eggplant, and maize plants found that lead accumulation was highest in the root structure in all plants [71].

As seen in Figure 2, the maize plant accumulated Cd metal in the order of roots > above-ground parts > soil. The high mobility of Cd metal compared to other metals facilitates its uptake by the plant [72]. In a study examining sorghum plants at different developmental stages (seedling and mature stage) under Cd stress separately, Cd accumulation was found to be highest in the roots of both plants [73]. In a similar study on lettuce, it was determined that Cd applied to the soil at different doses suppressed plant development and yield, and the metal accumulation level was higher than the allowed level for the plant and exceeded the acceptable limit [74,75]. Similarly, [76] found that more Cd and Pb accumulated in the roots of mustard grass (*Brassica juncea* L.) than in its shoots, and that cadmium accumulated more than lead.

### 3.5. Bioconcentration Factor and Translocation Factor

**Figure 3:**
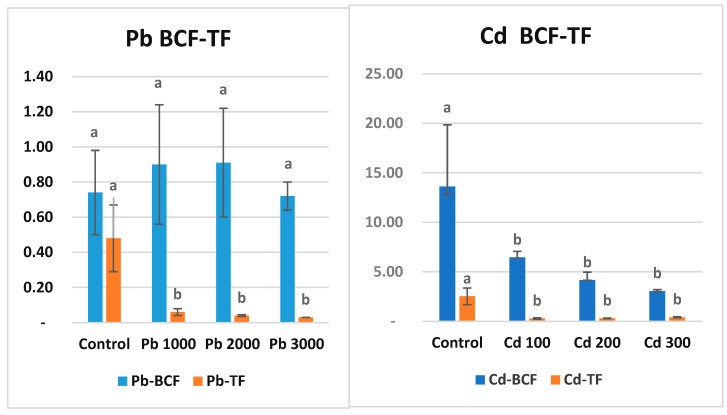
Bioconcentration factor (BCF) and translocation factor (TF) values (ppm) of Pb and Cd metals in maize after the 7-week growth period. There is no statistical difference between the averages shown with the same letter.

Bioconcentration factor (BCF) is the ratio of metal concentration in roots to soil. These parameters can be used as parameters to determine whether a plant can be classified as an accumulator. The value of translocation factors for heavy metals in a plant should be greater than one for it to be considered as a bioaccumulator [77,78]. In the present study, BCF Pb does not appear to be significantly different for all tested plants (Figure 3). All values are less than one. In light of these results, maize can be classified as an outlier for Pb metal (Figure 2). Cd content in soil was negatively correlated with the BCF of maize. It was observed that the BCF coefficient decreased with increasing Cd amount in soil. However, all BCFs in Cd treatments were in the same statistical group (*p* ˂ 0.05). BCF decreases with increasing concentrations of potentially toxic elements (PTE) in the soil because plants have a limited absorption capacity according to the pollutant, the medium becomes saturated, and the plant tissue concentration stabilizes [79]. In the present study, BCF, which was 6.45 in Cd1 application, decreased to 3.05 in Cd3 application. Translocation factor (TF) is the rate of metal transport from roots to shoots. TF values for Pb varied between 0.06 (Pb1) and 0.03 (Pb3). TF values for Cd varied between 0.27 (Cd1) and 0.40 (Cd3). This is expected for Cd, which accumulates the metal in its roots. TF values for both metals are less than one and are statistically in the same group (*p* ˂ 0.001). The higher accumulation of heavy metals in roots compared to shoots is due to the immobilization of metals from soil and sediments to the aboveground organs of plants [80]. Therefore, heavy metal BCF in roots is generally higher than that in shoots (stems and leaves) [81].

## 4. Discussion

Recent years have witnessed significant research into the toxicity of non-essential elements like Cd and Pb, primarily driven by escalating environmental pollution caused by these metals. Plants possess inherent defense mechanisms that effectively mitigate the stress induced by heavy metal exposure. These intrinsic tolerance mechanisms, extensively documented in the scientific literature, enable plants to thrive even in environments heavily contaminated with these metals [82].

Cadmium and Pb soil contamination poses a significant environmental challenge, demanding comprehensive and sustainable solutions. These toxic elements can originate from natural sources within the Earth’s crust or from human activities. Over time, cadmium and lead can accumulate and move within soil, increasing the risk of entering the food chain. This poses a serious threat to the health of living organisms throughout the food web [83].

The removal of heavy metals from soil using green plants is called phytoremediation. Phytoremediation is an emerging technology offering a potential solution for contaminated agricultural land and heavily polluted areas resulting from urban or industrial activities. Three primary strategies are currently employed to phytoextract inorganic substances from soil: (a) utilizing natural hyperaccumulators—plants with an inherent ability to accumulate high concentrations of contaminants; (b) enhancing uptake in high biomass species—stimulating contaminant uptake in plants with significant biomass through the application of chemical agents to the soil and plants; (c) phytovolatilization—transforming contaminants within the plant into volatile forms, facilitating their release into the atmosphere [84,85]. It has been shown that maize is an effective accumulator plant for phytoremediation of Cd- and Pb-polluted soils [86,87]

The findings of this study clearly indicate the detrimental effects of heavy metal applications on the growth and physiological characteristics of maize plants. Cadmium (Cd) and lead (Pb) were observed to cause significant growth retardation, with Cd exerting more pronounced negative effects compared to Pb. Under Cd300 treatment, plant height, fresh weight, dry weight, root fresh weight, and root dry weight decreased substantially. Pb treatments also resulted in growth reductions, but the impact was less severe than that of Cd (Table 1). Heavy metal toxicity led to a significant reduction in chlorophyll content and leaf area, inhibited protein synthesis, adversely affected photosynthesis, and impeded carbohydrate transport. This ultimately resulted in decreased biomass and disrupted root cell functioning [88]. These results confirm that while lead creates toxic effects in the roots and stems of plants, cadmium’s effects are more pronounced [48]. Studies have reported that heavy metal damage first appears in the roots of plants and that continued uptake of heavy metals adversely affects stem elongation [89,90]. This occurs as plants absorb heavy metals from the soil through their roots, accumulating them in the root tissues, which then disrupts root cell functioning. The findings indicate that heavy metal toxicity also leads to significant reductions in plant biomass. High concentrations of heavy metals inhibit protein formation in plant cells, adversely affect photosynthesis, and impede carbohydrate transport within the plant. It is documented in the literature that Cd inhibits plant growth by affecting cell wall expansion and cell division [91]. A study conducted on maize showed that increasing Cd concentrations (0.25, 0.50, and 0.75 mg kg^−1^) caused a gradual but significant decrease in various vegetative parameters such as germination rate, plant height, leaf number, and biomass. The highest Cd concentration had the most toxic effect on maize plants. Furthermore, leaf area measurements indicated a 13% reduction in Pb3000 treatments and a 59% reduction in Cd300 treatments. High levels of heavy metal exposure result in a general decrease in the morphophysiological characteristics of leaves, including leaf area [92]. The processes critical to leaf area expansion and overall plant growth, such as cell division and growth, are adversely affected by heavy metals like lead and cadmium [48,93]. Growth reduction occurs due to the increased accumulation of heavy metals in the vascular bundles of the stems [94]. Moreover, an increase in metal applications was observed to correspond with a decrease in leaf number. In lead applications, leaf number decreased by 9%, and in cadmium applications, it decreased by 20%. Researchers have indicated that the reduction in leaf number is due to the adverse effects of metals on water use efficiency [95].

Heavy metals, especially Cd, reduce the chlorophyll content in plants by inhibiting the enzymes involved in chlorophyll synthesis [96]. This study supports these findings by showing a significant decrease in chlorophyll content and is consistent with previous studies [97,98] (Table 2). The results of the study showed that heavy metal stress conditions increased EC and decreased LRWC (Table 2). The decrease in LRWC values may be due to the decrease in hydraulic conductivity caused by heavy metal toxicity [23]. These findings are consistent with other studies reporting similar effects of Cd and Pb applications on electrical conductivity and LRWC in maize plants [63,72]. Deterioration in water status was observed in plants exposed to different lead concentrations in various crops [20], including Helianthus annuus L. [99], wheat [100], and broad bean [101].

Heavy stress led to a general increase in antioxidant enzyme activity (Table 2). Under stress conditions, plants produce reactive oxygen species (ROS), toxic and reactive molecules that cause oxidative stress [102]. To mitigate cellular damage, plants activate antioxidant enzymes such as superoxide dismutase (SOD), catalase (CAT), ascorbate peroxidase (APX), and glutathione reductase (GR). Plant damage occurs when the capacity of these antioxidant mechanisms is overwhelmed [103]. Similarly, CAT activity increased in garden cress treated with Cd [94]. Higher SOD values in cadmium applications compared to lead applications can be attributed to higher tolerance to heavy metal stress [104].

Furthermore, heavy metal contamination increased reactive oxygen species (ROS) production, causing oxidative stress in maize plants (Table 2). This was evidenced by elevated levels of hydrogen peroxide (H_2_O_2_) and malondialdehyde (MDA), indicating cellular damage and lipid peroxidation. The study also revealed that Cd accumulated predominantly in the roots, with limited translocation to above-ground parts, classifying maize as an accumulator plant for Cd. The results of previous studies on maize are consistent with our findings [105,106]. Heavy metals induce the production of reactive oxygen species (ROS) in plants, leading to oxidative stress. ROS cause damage to various biomolecules in plant cells, such as DNA, proteins, and lipids [107,108]. Hydrogen peroxide (H_2_O_2_), a type of ROS, accumulates in plants in response to the toxic effects of heavy metals like Cd and Pb. H_2_O_2_ can cause lipid peroxidation in cell membranes, leading to cellular damage. Thus, the production of H_2_O_2_ is an indicator of oxidative stress caused by Cd and Pb [109,110]. Malondialdehyde (MDA) is a final product of lipid peroxidation and serves as an indicator of oxidative damage to cell membranes. Cd and Pb increase lipid peroxidation by attacking the lipid components of cell membranes, which leads to elevated MDA levels. MDA is used as a marker of cell membrane damage and overall oxidative stress [109,111,112].

The accumulation values of different Pb and Cd applications in soil, roots, and above-ground parts are shown in Figure 1. Lead metal is generally strongly retained in the soil, resulting in low phyto-availability, especially at high soil pH [113,114]. Due to Pb being immobilized in the soil, resilient plants can concentrate Pb in their roots, with very low translocation rates to the above-ground parts [115]. In this context, it is crucial to test metal-tolerant plant species for their ability to accumulate Pb in roots or translocate it to shoots. A study on maize investigated the accumulation of Cr, Pb, Ni, and Zn metals in plant organs. It was found that lead metal accumulated in the plant in the order of roots > leaves > stems > fruits [116]. Researchers have reported that maize is sensitive to heavy metals, with highly variable soil–plant metal transfer ratios and high extraction and bioaccumulation capacities [73,117]. The higher mobility of cadmium compared to other metals facilitates its uptake by plants [114]. A study examining cadmium accumulation at different growth stages (seedling and mature stages) in sorghum plants found the highest Cd accumulation rates in roots during both stages [104]. Another study on wheat determined that Cd accumulation follows the order of roots > shoots > grains [118].

Under Cd stress conditions, a study by [41] examined the root and shoot structure of 20 maize genotypes and found that the metal accumulates more in roots than in shoots. Additionally, it was reported that changes in root structure (such as root length, primary root depth, and average diameter) in maize plants grown in Cd-contaminated soil serve as a strategy to mitigate Cd toxicity and improve plant tolerance. Lower Cd content in shoots compared to roots and the Cd translocation factor indicate increased tolerance to Cd stress, suggesting that such genotypes may have production potential in moderately Cd-contaminated agricultural soils. [119] stated that the ability of maize roots to retain Cd contributes to reducing Cd accumulation in grains.

Some researchers have noted that plant responses to heavy metals can be categorized into exclusion and accumulation [120]. To determine whether a plant accumulates a metal, the bioconcentration factor (BCF) and translocation factor (TF) should be considered [121,122]. The BCF represents the ratio of heavy metal concentration in the plant roots to the concentration in the soil [40]. For a plant, the TF is the ratio of the average pollutant concentration in stems and leaves to that in roots, indicating the plant’s ability to transfer pollutants from roots to above-ground parts [123]. Plants with BCF > 1 and TF > 1 are termed accumulator plants; a high BCF indicates metal accumulation in roots, and a high TF indicates metal accumulation in above-ground organs. Excluder plants can either prevent metals from entering roots or restrict their translocation from roots to shoots (TF < 1) [124]. These plants absorb metals at low rates even when grown in high-metal-concentration soils [125]. In our study, Pb BCF and TF values were found to be less than 1 in all applications.

The bioconcentration factor (BCF) values for all cadmium (Cd) applications were found to be greater than 1. Specifically, BCF values greater than 2 are considered high and indicate potential plants for phytoremediation [123,126]. Researchers value plants that can absorb high amounts of Cd in their roots without translocating it to the shoots, as this minimizes risk to consumers [127]. The translocation factor (TF) values for all Cd applications were less than 1. In the control application, the TF value was greater than 1 due to the low metal density in the roots (Figure 2). Based on the BCF results, maize can be classified as an accumulator plant for Cd metal. Similar studies on maize have demonstrated that maize roots can absorb high amounts of Cd and sequester it within root cell vacuoles, thereby restricting its translocation to the shoots [54,128,129] (Figure 2).

### Recommendations

1. Phytoremediation Potential:
  - Given maize’s ability to accumulate Cd in its roots, further research should explore its potential use in phytoremediation strategies to clean Cd-contaminated soils.
  - Developing and utilizing metal-tolerant maize genotypes could enhance the efficiency of phytoremediation practices.
2. Agricultural Management:
  - Implementing soil amendments and chelating agents could mitigate heavy metal toxicity and improve plant growth and productivity in contaminated soils.
  - Regular monitoring and assessment of soil and plant metal concentrations could ensure food safety and minimize health risks.
3. Genetic Modification:
  - Genetic modification techniques could be explored to develop maize varieties with enhanced heavy metal tolerance and reduced translocation of metals to edible parts.
  - The underlying genetic and molecular mechanisms responsible for heavy metal uptake and detoxification in maize should be investigated.
4. Antioxidant Defense Mechanisms:
  - Research should examine enhancing antioxidant defense mechanisms in maize through breeding or biotechnological approaches to improve plant resilience to oxidative stress caused by heavy metal exposure.
5. Environmental and Health Implications:
  - Long-term studies should be conducted to assess the environmental and health implications of using maize for phytoremediation and the potential risks associated with heavy metal accumulation in food crops.
  - Sustainable agricultural practices that reduce heavy metal contamination and protect soil health should be promoted.

## 5. Conclusions

In conclusion, maize can be used as an effective accumulator plant for phytoremediation of Cd- and Pb-polluted soils. The variety used in this study, which is specific to the area, is promising for use as an accumulator. Furthermore, the study highlights the need for effective management strategies to mitigate the impact of heavy metal contamination on maize growth and productivity. Future research should focus on developing innovative solutions to enhance plant tolerance and improve crop resilience in contaminated environments.

## Figures and Tables

**Table 1:** Effects of heavy metal (Pb, Cd) applications on some plant growth characteristics in maize plants (mean ± standard deviation) ^1^.

Treatments mg kg^−1^	Plant Height (cm)	Plant Fresh Weight (g)	Plant Dry Weight (g)	Root Fresh Weight (g)	Root Dry Weight (g)	Stem Diameter (mm)	Number of Leaves (per Plant)	Leaf Area (cm^2^/Plant)
**Control**	54.96 ± 1.017 a	17.27 ± 0.754 a	4.91 ± 0.046 a	13.49 ± 1.679 a	1.28 ± 0.089 a	8.12 ± 0.448 a	6.66 ± 0.0667 a	285.19 ± 2.777 a
**Pb 1000**	53.60 ± 2.354 a	16.05 ± 0.173 a	4.82 ± 0.069 a	13.35 ± 1.441 a	1.25 ± 0.124 a	6.34 ± 0.246 b	6.26 ± 0.0667 ab	304.28 ± 49.023 a
**Pb 2000**	49.33 ± 1.212 b	15.65 ± 1.224 a	3.83 ± 0.321 b	11.81 ± 1.247 a	1.22 ± 0.029 a	6.27 ± 0.202 b	6.26 ± 0.0667 ab	258.57 ± 14.929 a
**Pb 3000**	43.80 ± 0.953 c	14.60 ± 0.678 a	3.68 ± 0.131 b	11.18 ± 0.580 a	1.15 ± 0.816 a	6.11 ± 0.096 bc	6.06 ± 0.0667 b	249.27 ± 19.837 a
**Cd 100**	39.26 ± 0.3712 d	9.83 ± 0.985 b	3.13 ± 0.550 b	7.21 ± 0.765 b	0.77 ± 0.531 b	5.40 ± 0.178 cd	5.93 ± 0.0667 b	230.82 ± 20.141 a
**Cd 200**	34.86 ± 1.185 e	8.19 ± 1.364 bc	2.12 ± 0.221 c	7.11 ± 0.556 b	0.73 ± 0.047 b	4.79 ± 0.119 d	5.86 ± 0.133 b	148.63 ± 20.005 b
**Cd 300**	31.93 ± 1.313 e	6.28 ± 0.416 c	1.61 ± 0.121 c	5.92 ± 0.003 b	0.60 ± 0.011 b	3.92 ± 0.258 e	5.33 ± 0.333 c	116.15 ± 11.787 b
*p*	**	**	**	**	**	**	*	**

^1^ Each value is the mean of three replicates ± SD. Data followed by a different letter in column were significantly different according to the DMRT. ** *p* < 0.01, * *p* < 0.05. Pb: lead, Cd: cadmium.

**Table 2:** Effects of heavy metal (Pb, Cd) applications on physiological properties and antioxidant enzyme activities in maize plants (mean ± standard deviation) ^1^.

Treatments (mg kg^−1^)	Chlorophyll Reading Value (SPAD)	EC (%)	LRWC (%)	CAT-(EU/gFW)	POD-(EU/gFW)	SOD-(EU/gFW)	H_2_O_2_ (mmol kg^−1^)	MDA-(nmol g^−1^)
**Control**	46.28 ± 0.227 a	10.76 ± 0.509 c	81.48 ± 0.239 a	0.02 ± 0.001 b	8.21 ± 0.001 d	76.72 ± 0.341 c	98.69 ± 1.008 e	1.75 ± 0.033 f
**Pb 1000**	46.14 ± 0.682 a	12.58 ± 0.737 c	70.58 ± 0.398 c	0.02 ± 0.002 b	7.34 ± 0.016 e	82.56 ± 0.828 b	146.66 ± 1.118 b	1.90 ± 0.086 f
**Pb 2000**	45.01 ± 0.724 ab	14.80 ± 0.655 bc	65.05 ± 1.544 de	0.008 ± 0.000 c	10.98 ± 0.179 b	82.76 ± 2.146 b	147.29 ± 0.454 ab	2.20 ± 0.036 e
**Pb 3000**	43.76 ± 0.283 b	15.22 ± 0.052 bc	62.32 ± 0.516 e	0.017 ± 0.001 b	7.36 ± 0.084 e	82.13 ± 1.815 b	152.57 ± 0.238 a	2.46 ± 0.040 d
**Cd 100**	38.97 ± 0.377 c	17.51 ± 3.291 ab	75.40 ± 1.076 b	0.013 ± 0.001 bc	14.06 ± 0.414 a	87.00 ± 0.716 a	115.82 ± 2.788 d	5.68 ± 0.136 c
**Cd 200**	37.04 ± 0.203 d	18.64 ± 0.192 ab	72.68 ± 0.664 bc	0.015 ± 0.002 b	8.86 ± 0.126 c	80.53 ± 0.249 bc	118.25 ± 0.158 d	6.11 ± 0.073 b
**Cd 300**	36.09 ± 0.456 d	20.93 ± 1.761 a	66.41 ± 1.772 d	0.032 ± 0.002 a	7.13 ± 0.093 e	89.70 ± 1.735 a	125.68 ± 3.682 c	6.46 ± 0.019 a
*p*	**	*	**	**	**	**	**	**

^1^ Each value is the mean of three replicates ± SD. Data followed by a different letter in column were significantly different according to the DMRT. ** *p* < 0.01, * *p* < 0.05. Pb: lead, Cd: cadmium, EC: electrolyte conductivity, LRWC: leaf relative water content, CAT: catalase, POD: peroxidase, SOD: superoxide dismutase, H_2_O_2_: hydrogen peroxide, MDA: malondialdehyde.

## Data Availability

The original contributions presented in this study are included in the article. Further inquiries can be directed to the corresponding author.

## References

[1] Xu D.M., Fu R.B., Tong Y.H., Shen D.L., Guo X.P. (2021). The potential environmental risk implications of heavy metals based on their geochemical and mineralogical characteristics in the size-segregated zinc smelting slags. J. Clean. Prod..

[2] Hu Y., Liu X., Bai J., Shih K., Zeng E.Y., Cheng H. (2013). Assessing heavy metal pollution in the surface soils of a region that had undergone three decades of intense industrialization and urbanization. Environ. Sci. Pollut. Res..

[3] Nnaji N.D., Onyeaka H., Miri T., Ugwa C. (2023). Bioaccumulation for heavy metal removal: A review. Appl. Sci..

[4] Atasoy N. (2024). Atık Sulardan Ağır Metal Giderimi. J. Inst. Sci. Technol..

[5] Clemens S. (2006). Toxic metal accumulation, responses to exposure and mechanisms of tolerance in plants. Biochimie.

[6] Jomova K., Alomar S.Y., Nepovimova E., Kuca K., Valko M. (2024). Heavy metals: Toxicity and human health effects. Arch. Toxicol..

[7] Bermudez G.M.A., Jasan R., Pla R., Pignata M.L. (2012). Heavy metals and trace elements in atmospheric fall-out: Their relationship with topsoil and wheat element composition. J. Hazard. Mater..

[8] Yaldız G., Şekeroğlu N. (2013). Tıbbi ve aromatik bitkilerin bazı ağır metallere tepkisi. Türk Bilimsel Derlemeler Derg..

[9] Yılmaz H.Ş. (2019). Farklı ağır metal uygulamalarının tane sorgum çeşitlerinde ağır metal birikimi, morfolojik ve yem kalite özelliklerine etkisi. Doctoral Thesis.

[10] Sanaei S., Sadeghinia M., Meftahizade H., Ardakani A.F., Ghorbanpour M. (2022). Cadmium and lead differentially affect growth, physiology, and metal accumulation in guar (*Cyamopsis tetragonoloba* L.) genotypes. Environ. Sci. Pollut. Res..

[11] Gallo-Franco J.J., Sosa C.C., Ghneim-Herrera T., Quimbaya M. (2020). Epigenetic control of plant response to heavy metal stress: A new view on aluminum tolerance. Front. Plant Sci..

[12] Noor I., Sohail H., Sun J., Nawaz M.A., Li G., Hasanuzzaman M., Liu J. (2022). Heavy metal and metalloid toxicity in horticultural plants: Tolerance mechanism and remediation strategies. Chemosphere.

[13] Thakur M., Praveen S., Divte P.R., Mitra R., Kumar M., Gupta C.K., Singh B. (2022). Metal tolerance in plants: Molecular and physicochemical interface determines the “not so heavy effect” of heavy metals. Chemosphere.

[14] Cao Y., Tan Q., Zhang F., Ma C., Xiao J., Chen G. (2022). Phytoremediation potential evaluation of multiple Salix clones for heavy metals (Cd, Zn and Pb) in flooded soils. Sci. Total Environ..

[15] Çılgın K. (2022). Buğday (*Triticum Aestivum* L.) ve Arpa (*Hordeum Vulgare* L.) Bitkilerinde Kadmiyum Stresine Bağlı Genotoksik Etkilerin Belirlenmesi. Master’s Thesis.

[16] Wang L., Zheng B., Yuan Y., Xu Q., Chen P. (2020). Transcriptome profiling of Fagopyrum tataricum leaves in response to lead stress. BMC Plant Biol..

[17] Zhang L., Zhu Y., Gu H., Lam S.S., Chen X., Sonne C., Peng W. (2024). A review of phytoremediation of environmental lead (pb) contamination. Chemosphere.

[18] Zhang Y., Li T., Guo Z., Xie H., Hu Z., Ran H., Jiang Z. (2023). Spatial heterogeneity and source apportionment of soil metal (loid) s in an abandoned lead/zinc smelter. J. Environ. Sci..

[19] Gong X., Yang F., Pan X., Shao J. (2023). Accumulation of silicon in shoots is required for reducing lead uptake in rice. Crop J..

[20] Collin S., Baskar A., Geevarghese D.M., Ali M.N.V.S., Bahubali P., Choudhary R., Lvov V., Tovar G.I., Senatov F., Koppala S. (2022). Bioaccumulation of lead (Pb) and its effects in plants: A review. J. Hazard. Mater. Lett..

[21] Pourrut B., Ramos I., Esteban E., Lucena J.J., Garate A. (2002). Cadmium uptake and sub-cellular distribution in plants os *Lactuca* sp.. Cd- Mn Interaction. Plant Sci..

[22] Gupta D.K., Nicoloso F.T., Schetinger M.R.C., Rossato L.V., Pereira L.B., Castro G.Y., Srivastava S., Tripathi R.D. (2009). Defense mechanism in hydroponically grown Zea mays seedlings under moderate lead stress. J. Hazard. Mater..

[23] Yoon J., Cao X., Zhou Q., Ma L.Q. (2006). Accumulation of Pb, Cu, and Zn in native plants growing on a contaminated Florida site. Sci. Total Environ..

[24] Qu F., Zheng W. (2024). Cadmium Exposure: Mechanisms and Pathways of Toxicity and Implications for Human Health. Toxics.

[25] Hussain C.M., Kecili R. (2019). Modern Environmental Analysis Techniques for Pollutants.

[26] Tüver G. (2021). Farklı Sulama Seviyesi Koşullarında Kadmiyumun Fındık Turpunda (*Raphanus sativus* L.) Bitki Gelişimi Üzerine Etkisi. Yüksek Lisans Tezi.

[27] Ali H., Khan E. (2018). Trophic transfer, bioaccumulation, and biomagnification of non-essential hazardous heavy metals and metalloids in food chains/webs—Concepts and implications for wildlife and human health. Human. Ecol. Risk Assess. An. Int. J..

[28] Simmler M., Ciadamidaro L., Schulin R., Madejon P., Reiser R., Clucas L., Weber P., Robinson B. (2013). Lignite reduces the solubility and plant uptake of cadmium in pasturelands. Environ. Sci. Technol..

[29] Li T., Chang Q., Yuan X., Li J., Ayoko G.A., Frost R., Che H., Zhang X., Song Y., Song W. (2017). Cadmium transfer from contaminated soils to the human body through rice consumption in southern Jiangsu Province, China Environ. Sci. J. Tegr. Environ. Res. Process. Impacts.

[30] Ahmad P., Abd Allah E.F., Hashem A., Sarwat M., Gucel S. (2016). Exogenous application of selenium mitigates cadmium toxicity in *Brassica juncea* L. (Czern & Cross) by up-regulating antioxidative system and secondary metabolites. J. Plant Growth Regul..

[31] Jali P., Pradhan C., Das A.B. (2016). Effects of cadmium toxicity in plants: A Review. Artic. Sch. Acad. J. Biosci..

[32] Vezza M.E., Llanes A., Travaglia C., Agostini E., Talano M.A. (2018). Arsenic stress effects on root water absorption in soybean plants: Physiological and morphological aspects. Plant Physiol. Biochem..

[33] Aslam M., Aslam A., Sheraz M., Ali B., Ulhassan Z., Najeeb U., Gill R.A. (2021). Lead toxicity in cereals: Mechanistic insight into toxicity, mode of action, and management. Front. Plant Sci..

[34] Vasilachi I.C., Stoleru V., Gavrilescu M. (2023). Analysis of heavy metal impacts on cereal crop growth and development in contaminated soils. Agriculture.

[35] ATSDR (2022). Konu: Agency for Toxic Substances and Disease Registry (ATSDR)’s Substance Priority List. https://www.atsdr.cdc.gov/programs/substance-priority-list.html?CDC_AAref_Val=https://www.atsdr.cdc.gov/spl/index.html.

[36] Amanjyoti, Singh J., Sowdhanya D., Rasane P., Singh J., Ercisli S., Ullah R. (2024). Maize. Cereals and Nutraceuticals.

[37] FAO (2022). Crop Statistics. http://www.fao.org/faostat/en/.

[38] OECD, FAO (2019). OECD-FAO Agricultural Outlook 2019–2018.

[39] Figlioli F., Sorrentino M.C., Memoli V., Arena C., Maisto G., Giordano S., Spagnuolo V. (2019). Overall plant responses to Cd and Pb metal stress in maize: Growth pattern, ultrastructure, and photosynthetic activity. Environ. Sci. Pollut. Res..

[40] Jiang L., Yi X., Xu B., Wang W., Lai K. (2019). Soil treatment and crop rotation for in situ remediation of heavy metal-contaminated agricultural soil in gold mining areas. Hum. Ecol. Risk Assess. Int. J..

[41] Wu Y., An T., Gao Y., Kuang Q., Liu S., Liang L., Bingcheng X., Suiqi Z., Deng X., Chen Y. (2023). Genotypic variation in the tolerance to moderate cadmium toxicity among 20 maize genotypes with contrasting root systems. J. Sci. Food Agric..

[42] Ren C., Xiao J.H., Li J.T., Du Q.Q., Zhu L.W., Wang H., Zhao H.Y. (2022). Accumulation and Transport Characteristics of Cd, Pb, Zn, and As in Different Maize Varieties. Huan Jing Ke Xue=Huanjing Kexue.

[43] Sevgi K., Leblebici S. (2022). Bitkilerde ağır metal stresine verilen fizyolojik ve moleküler yanıtlar. J. Anatol. Environ. Anim. Sci..

[44] Singh S., Parihar P., Singh R., Singh V.P., Prasad S.M. (2016). Heavy metal tolerance in plants: Role of transcriptomics, proteomics, metabolomics, and ionomics. Front. Plant Sci..

[45] Li S., Han X., Lu Z., Qiu W., Yu M., Li H., He Z., Zhuo R. (2022). MAPK Cascades and Transcriptional Factors: Regulation of Heavy Metal Tolerance in Plants. Int. J. Mol. Sci..

[46] Tan M. (2018). Baklagil ve Buğdaygil Yem Bitkileri Kitabı.

[47] Ekinci M., Yildirim E., Ağar G., Yüksel E.A., Aydin M., Örs S., Kul R. (2023). Determination of cadmium and/or drought stress effects on some plant phytohormone contents and hormone gene expressions in bean (*Phaseolus vulgaris* L.). Turk. J. Agric. For..

[48] Yildirim E., Ekinci M., Turan M., Güleray A.G.A.R., Selda Ö.R.S., Dursun A., Balci T. (2019). Impact of cadmium and lead heavy metal stress on plant growth and physiology of rocket (*Eruca sativa* L.). Kahramanmaraş Sütçü İmam Üniversitesi Tarım Ve Doğa Derg.

[49] Yildirim E., Karlidag H., Turan M. (2009). Mitigation of salt stress in strawberry by foliar K, Ca and Mg nutrient supply. Plant Soil Environ..

[50] (2025). https://media.licdn.com/dms/document/media/v2/D4D1FAQHCx5U2EMVjyg/feedshare-document-pdf-analyzed/feedshare-document-pdf-analyzed/0/1682363438103?e=1740614400&v=beta&t=WVGRLlAKH8PiAvCFtmuGnBq31QRDB2NlVdJYRZha9kc.

[51] Turan M., Ekinci M., Kul R., Boynueyri F.G., Yildirim E. (2022). Mitigation of salinity stress in cucumber seedlings by exogenous hydrogen sulfide. J. Plant Res..

[52] Ors S., Ekinci M., Yildirim E., Sahin U., Turan M., Dursun A. (2021). Interactive effects of salinity and drought stress on photosynthetic characteristics and physiology of tomato (*Lycopersicon esculentum* L.) seedlings. S. Afr. J. Bot..

[53] Liu S., Dong Y., Xu L., Kong J. (2014). Effects of foliar applications of nitric oxide and salicylic acid on salt-induced changes in photosynthesis and antioxidative metabolism of cotton seedlings. Plant Growth Regul..

[54] Sharmin S., Lipka U., Polle A., Eckert C. (2021). The influence of transpiration on foliar accumulation of salt and nutrients under salinity in poplar (Populus × canescens). PLoS ONE.

[55] Yildirim E., Ekinci M., Turan M., Dursun A., Kul R., Parlakova F. (2015). Roles of glycine betaine in mitigating deleterious effect of salt stress on lettuce (*Lactuca sativa* L.). Arch. Agron. Soil. Sci..

[56] González L., González-Vilar M. (2001). Determination of Relative Water Content. Handbook of Plant Ecophysiology Techniques.

[57] Ma H., Zhao C., Zhang L., Liu Z., Zhang F., Wang H., Peng M. (2023). Bioavailability, Sources, and Transfer Behavior of Heavy Metals in Soil–Crop Systems from a High Geological Background Area Impacted by Artisanal Zn Smelting in Guizhou Province, Southwest China. Processes.

[58] Sahin U., Ekinci M., Ors S., Turan M., Yildiz S., Yildirim E. (2018). Effects of individual and combined effects of salinity and drought on physiological, nutritional and bio-chemical properties of cabbage (Brassica oleracea var. capitata). Sci. Hortic..

[59] Rizvi A., Khan M.S. (2018). Heavy metal induced oxidative damage and root morphology alterations of maize (*Zea mays* L.) plants and stress mitigation by metal tolerant nitrogen fixing Azotobacter chroococcum. Ecotoxicol. Environ. Saf..

[60] Shahzad A., Qin M., Elahie M., Naeem M., Bashir T., Yasmin H., Younas M., Aree A., Irfan M., Billah M. (2021). Bacillus pumilus induced tolerance of Maize (*Zea mays* L.) against Cadmium (Cd) stress. Sci. Rep..

[61] Gidlow D.A. (2004). Lead toxicity. Occup. Med..

[62] Yerli C., Çakmakçı T., Şahin Ü., Tüfenkçi Ş. (2020). Ağır Metallerin Toprak, Bitki, su ve Insan Sağlığına Etkileri.

[63] Alyemeni M.N., Ahanger M.A., Wijaya L., Alam P., Ahmad P. (2017). Contrasting Tolerance Among SoybeanGenotypes Subjected to Different Levels of Cadmium Stress. Pak. J. Bot..

[64] Manousaki E., Kalogerakis N. (2009). Phytoextraction of Pb and Cd by the Mediterranean saltbush (*Atriplex halimus* L.): Metal Uptake in Relation to Salinity. Environ. Sci. Pollut. R..

[65] Ahmad P., Nabi G., Ashraf M. (2011). Cadmium-Induced Oxidative Damage in Mustard [*Brassica juncea* L.) Czern.& Coss.] Plants can be Alleviated by Salicylic Acid. S. Afr. J. Bot..

[66] AbdElgawad H., Zinta G., Hegab M.M., Pandey R., Asard H., Abuelsoud W. (2016). Yüksek tuzluluk, mısır fidelerinin organlarında farklı oksidatif stres ve antioksidan tepkilere neden olur. Front. Plant Sci..

[67] Engwa G.A., Ferdinand P.U., Nwalo F.N., Unachukwu M.N. (2019). Mechanism and health effects of heavy metal toxicity in humans. Poisoning in the Modern World-New Tricks for an Old Dog?.

[68] Wang S., Shi X., Salam M.M.A., Chen G. (2021). Integrated study on subcellular localization and chemical speciation of Pb reveals root strategies for Pb sequestration and detoxification in Salix integra. Plant Soil.

[69] Kumar B., Smita K., Flores L.C. (2017). Plant mediated detoxification of mercury and lead. Arab. J. Chem..

[70] Labrecque M., Teodorescu T.I., Daigle S. (1995). Effect of wastewater sludge on growth and heavy metal bioaccumulation of two Salix species. Plant Soil.

[71] Kafadar F., Saygıdeğer S. (2010). Gaziantep İlinde Organize Sanayi Bölgesi Atık Suları İle Sulanan Bazı Tarım Bitkilerinde Kurşun Miktarlarının Belirlenmesi. Ekoloji.

[72] Kabata-Pendias A. (2011). Trace Elements in Soils and Plants.

[73] Jia W., Lv S., Feng J., Li J., Li Y., Li S. (2016). Morphophysiological characteristic analysis demonstrated the potential of sweet sorghum (*Sorghum bicolor* (L.) Moench) in the phytoremediation of cadmium-contaminated soils. Environ. Sci. Pollut. Res..

[74] Loi N.N., Sanzharova N.I., Shchagina N.I., Mironova M.P. (2018). The effect of cadmium toxicity on the development of Lettuce plants on contaminated sod-podzolic soil. Russ. Agric. Sci..

[75] Pereira B.F.F., Rozane D.E., Araújo S.R., Barth G., Queiroz R.J.B., Nogueira T.A.R., Malavolta E. (2011). Cadmium availability and accumulation by lettuce and rice. Rev. Bras. De Ciência Do Solo.

[76] John R., Ahmad P., Gadgil K., Sharma S. (2009). Heavy metal toxicity: Effect on plant growth, biochemical parameters and metal accumulation by *Brassica juncea* L.. Int. J. Plant Prod..

[77] Takarina N.D., Pin T.G. (2017). Bioconcentration factor (BCF) and translocation factor (TF) of heavy metals in mangrove trees of Blanakan fish farm. Makara J. Sci..

[78] Sürmen B., Kılıç D.D., Kutbay H.G., Tuna E.E. (2019). Doğal olarak yayılış gösteren *Lepidium draba* L. türünün fitoremediasyon yönteminde kullanılabilirliğinin araştırılması. Avrupa Bilim. Teknol. Derg..

[79] McLaughlin M.J., Smolders E., Degryse F., Rietra R. (2011). Uptake of metals from soil into vegetables. Dealing with Contaminated Sites: From Theory Towards Practical Application.

[80] Hidayati N. (2013). Mekanisme fisiologis tumbuhan hiperakumulator logam berat. J. Teknol. Lingkung..

[81] Hidayati N., Rini D.S.R. Assessment on Lead and Cadmium Bioaccumulators for Phytoremediation Contaminated Rice Fields in Bekasi Districts, West Java. Proceedings of the 9th International Symposium for Sustainable Humanosphere (ISSH): “Integrated Smart Technology and Society for Sustainable Humanosphere.

[82] Wierzbicka M.H., Przedpełska E., Ruzik R., Ouerdane L., Połeć-Pawlak K., Jarosz M., Szakiel A. (2007). Comparison of the toxicity and distribution of cadmium and lead in plant cells. Protoplasma.

[83] Bouida L., Rafatullah M., Kerrouche A., Qutob M., Alosaimi A.M., Alorfi H.S., Hussein M.A. (2022). A review on cadmium and lead contamination: Sources, fate, mechanism, health effects and remediation methods. Water.

[84] Mojiri A. (2011). The potential of corn (Zea mays) for phytoremediation of soil contaminated with cadmium and lead. J. Biol. Environ. Sci..

[85] Poniedziałek M., Sękara A., Jędrszczyk E., Ciura J. (2010). Phytoremediation efficiency of crop plants in removing cadmium, lead and zinc from soil. Folia Hortic..

[86] Azizian A., Amin S., Maftoun M., Emam Y., Noshadi M. (2013). Response of Corn to Cadmium and Drought Stress and Its Potential Use for Phytoremediation. J. Agric. Sci. Technol..

[87] Cheng S.F., Huang C.Y., Lin Y.C., Lin S.C., Chen K.L. (2015). Phytoremediation of lead using corn in contaminated agricultural land—An in situ study and benefit assessment. Ecotoxicol. Environ. Saf..

[88] Yang Y., Wang S., Zhao C., Jiang X., Gao D. (2024). Responses of non-structural carbohydrates and biomass in plant to heavy metal treatment. Sci. Total Environ..

[89] Manivasagaperumal R., Vijayarengan P., Balamurugan S., Thiyagarajan G. (2011). Effect of copper on growth, dry matter yield and nutrient content of *Vigna radiata* (L.) Wilczek. J. Phytol..

[90] Marschner H. (2011). Marschner’s Mineral Nutrition of Higher Plants.

[91] Goncharuk E.A., Zagoskina N.V. (2023). Heavy metals, their phytotoxicity, and the role of phenolic antioxidants in plant stress responses with focus on cadmium. Molecules.

[92] Nagajyoti P., Lee K., Sreekanth T. (2010). Heavy metals, occurrence and toxicity for plants: A review. Environ. Chem. Lett..

[93] Ehlert C., Maurel C., Tardieu F., Simonneau T. (2009). Aquaporin-Mediated Reduction in Maize Root Hydraulic Conductivity Impacts Cell Turgor and Leaf Elongation even without Changing Transpiration. Plant Physiol..

[94] Rizvi A., Khan M.S. (2019). Heavy metal-mediated toxicity to maize: Oxidative damage, antioxidant defence response and metal distribution in plant organs. Int. J. Environ. Sci. Technol..

[95] Hatamian M., Nejad A.R., Kafi M., Souri M.K., Shahbazi K. (2020). Nitrate improves hackberry seedling growth under cadmium application. Heliyon.

[96] Poschenrieder C., Barceló J., Prasad M.N.V. (2004). Water relations in heavy metal stressed plants. Heavy Metal Stress in Plants.

[97] Fan S.X., Zhang N., Sun M.H., Hou X.D. (2024). Screening and stress responsive characteristics of potential hyperaccumulator of Pb, Zn, and Cd compound heavy metals. Huan Jing Ke Xue Huanjing Kexue.

[98] Phaenark C., Seechanhoi P., Sawangproh W. (2024). Metal toxicity in Bryum coronatum Schwaegrichen: Impact on chlorophyll content, lamina cell structure, and metal accumulation. Int. J. Phytoremediat..

[99] Kastori R., Petrovic M., Petrovic N. (2008). Effect of excess lead, cadmium, copper, and zinc on water relations in sunflower. J. Plant Nutr..

[100] Alsokari S.S., Aldesuquy H.S. (2011). Synergistic effect of polyamines and waste water on leaf turgidity, heavy metals accumulation in relation to grain yield. J. Appl. Sci. Res..

[101] Pourrut B. (2008). Implication du Stress Oxydatif Dans la Toxicité du Plomb sur une Plante Modèle, Vicia faba. Ph.D. Thesis.

[102] Yildirim E., Ekinci M., Turan M., Ağar G., Dursun A., Kul R., Argin S. (2021). Humic + Fulvic acid mitigated Cd adverse effects on plant growth, physiology and biochemical properties of garden cress. Sci. Rep..

[103] Hussain I., Iqbal M., Qurat-Ul-Ain S., Rasheed R., Mahmood S., Perveen A., Wahid A. (2012). Cadmium dose and exposure-time dependent alterations in growth and physiology of maize (Zea mays). Int. J. Agric. Biol..

[104] Yang W.W., Lıu M., Cao M.Z., Zhang C.L. (2014). Accumulation and transfer of lead (Pb) and cadmium (Cd) on different species of maize. J. Ecol. Rural. Environ..

[105] Sofy M.R., Seleiman M.F., Alhammad B.A., Alharbi B.M., Mohamed H.I. (2020). Mini-mizing adverse effects of pb on maize plants by combined treatment with jasmonic, salicylic acids and proline. Agronomy.

[106] Anjum S.A., Tanveer M., Hussain S., Bao M., Wang L., Khan I., Shahzad B. (2015). Cadmium toxicity in Maize (*Zea mays* L.): Consequences on antioxidative systems, reactive oxygen species and cadmium accumulation. Environ. Sci. Pollut. Res..

[107] Gill S.S., Tuteja N. (2010). Reactive oxygen species and antioxidant machinery in abiotic stress tolerance in crop plants. Plant Physiol. Biochem..

[108] Apel K., Hirt H. (2004). Reactive oxygen species: Metabolism, oxidative stress, and signal transduction. Annu. Rev. Plant Biol..

[109] Gu J., Hu C., Jia X., Ren Y., Su D., He J. (2022). Physiological and biochemical bases of spermidine-induced alleviation of cadmium and lead combined stress in rice. Plant Physiol. Biochem..

[110] Bamagoos A.A., Alharby H.F., Abbas G. (2022). Differential uptake and translocation of cadmium and lead by quinoa: A multivariate comparison of physiological and oxidative stress responses. Toxics.

[111] Zhu Y., Dong Y., Zhu N., Jin H. (2022). Foliar application of biosynthetic nano-selenium alleviates the toxicity of Cd, Pb, and Hg in Brassica chinensis by inhibiting heavy metal adsorption and improving antioxidant system in plant. Ecotoxicol. Environ. Saf..

[112] Giannakoula A., Therios I., Chatzissavvidis C. (2021). Effect of lead and copper on photosynthetic apparatus in citrus (*Citrus aurantium* L.) plants. The role of antioxidants in oxidative damage as a response to heavy metal stress. Plants.

[113] Salazar M.J., Pignata M.L. (2014). Lead accumulation in plants grown in polluted soils. Screening of native species for phytoremediation. J. Geochem. Explor. Tion.

[114] Lu Y., Yao H., Shan D., Jiang Y., Zhang S., Yang J. (2015). Heavy metal residues in soil and accumulation in maize at long-term wastewater irrigation area in Tongliao, China. J. Chem..

[115] Adewole M.B., Oyebanji B.O., Igbekele K. (2019). Phytoremediation potential of two maize varieties cultivated on metal-particulate-contaminated soil. Ghana J. Agric. Sci..

[116] Aladesanmi O.T., Oroboade J.G., Osisiogu C.P., Osewole A.O. (2019). Bioaccumulation factor of selected HMs in *Zea mays*. J. Health Pollut..

[117] Retamal-Salgado J., Hirzel J., Walter I., Matus I. (2017). Bioabsorption and bioaccumulation of cadmium in the straw and grain of maize (*Zea mays* L.) in growing soils contaminated with cadmium in different environment. Int. J. Environ. Res. Public Health.

[118] Rascio N., Navari-Izzo F. (2011). Heavy metal hyperaccumulating plants: How and why do they do it? And what makes them so interesting?. Plant Sci..

[119] Chen Z., Ai Y., Fang C., Wang K., Li W., Liu S., Li C., Xiao J., Huang Z. (2014). Distribution and phytoavailability of heavy metal chemical fractions in artificial soil on rock cut slopes alongside railways. J. Hazard. Mater..

[120] Wahsha M., Bini C., Argese E., Minello F., Fontana S., Wahsheh H. (2012). Heavy metals accumulation in willows growing on Spolic Technosols from the abandoned Im-perina Valley mine in Italy. J. Geochem. Explor..

[121] Madanan M.T., Shah I.K., Varghese G.K., Kaushal R.K. (2021). Application of Aztec Marigold (*Tagetes erecta* L.) for phytoremediation of heavy metal polluted lateritic soil. Environ. Chem. Ecotoxicol..

[122] Boularbah A., Schwartz C., Bitton G., Aboudrar W., Ouhammou A., Morel J.L. (2006). Heavy metal contamination from mining sites in South Morocco: 2. Assessment of metal accumulation and toxicity in plants. Chemosphere.

[123] Marchiol L., Sacco P., Assolari S., Zerbi G. (2004). Reclamation of polluted soil: Phytore-mediation potential of crop-related Brassica species. Water Air Soil. Pollut..

[124] Pasricha S., Mathur V., Garg A., Lenka S., Verma K., Agarwal S. (2021). Molecular mechanisms underlying heavy metal uptake, translocation and tolerance in hyperaccumulators-an analysis: Heavy metal tolerance in hyperaccumulators. Environ. Chall..

[125] Rezapour S., Atashpaz B., Moghaddam S.S., Kalavrouziotis I.K., Damalas C.A. (2019). Cadmium accumulation, translocation factor, and health risk potential in a wastewater-irrigated soil-wheat (*Triticum aestivum* L.) system. Chemosphere.

[126] Cobbett C.S. (2000). Phytochelatins and their roles in heavy metal detoxification. Plant Physiol..

[127] Adriano D.C. (2001). Trace Elements in Terrestrial Environments: Biogeochemistry, Bioavailability, and Risks of Metals.

[128] Ak A., Yücel E. (2011). Ecotoxicological effects of heavy metal stress on antioxidant en-zyme levels of triticum aestivum cv. Alpu. Biol. Divers. Conserv..

[129] Bhaduri A.M., Fulekar M.H. (2012). Antioxidant Enzyme Responses of Plants to Heavy Metal Stress. Rev. Environ. Sci. Bio-Technol..

